# International school-related sedentary behaviour recommendations for children and youth

**DOI:** 10.1186/s12966-022-01259-3

**Published:** 2022-04-05

**Authors:** Travis J. Saunders, Scott Rollo, Nicholas Kuzik, Iryna Demchenko, Stacey Bélanger, Kara Brisson-Boivin, Valerie Carson, Bruno G. G. da Costa, Melanie Davis, Susan Hornby, Wendy Yajun Huang, Barbi Law, Michelle Ponti, Chris Markham, Jo Salmon, Jennifer R. Tomasone, Antonius J. Van Rooij, Lucy-Joy Wachira, Katrien Wijndaele, Mark S. Tremblay

**Affiliations:** 1 Department of Applied Human Sciences, University of Prince Edward Island, Charlottetown, Canada; 2 Healthy Active Living and Obesity Research Group, Children’s Hospital of Eastern Ontario Research Institute, Ottawa, Canada; 3 Digital Health Task Force, Canadian Paediatric Society, Ottawa, Canada; 4 Mediasmarts, Ottawa, Canada; 5 Faculty of Kinesiology, Sport, and Recreation, University of Alberta, Edmonton, Canada; 6 School of Physical and Health Education, Nipissing University, North Bay, Ontario Canada; 7 Physical and Health Education Canada, Ottawa, Canada; 8 Pan-Canadian Joint Consortium for School Health Secretariat, Summerside, Canada; 9 Department of Sport, Physical Education and Health, Hong Kong Baptist University, Hong Kong, China; 10 Ontario Physical and Health Education Association, Ottawa, Canada; 11 Institute for Physical Activity and Nutrition, Deakin University, Geelong, Australia; 12 School of Kinesiology and Health Studies, Queen’s University, Kingston, Ontario Canada; 13 Department of Youth, Trimbos Institute, Utrecht, The Netherlands; 14 Physical Education, Exercise and Sports Science, Kenyatta University, Nairobi, Kenya; 15 MRC Epidemiology Unit, University of Cambridge, Cambridge, UK; 16 Department of Pediatrics, University of Ottawa, Ottawa, Canada; 17 Department of Health Sciences, Carleton University, Ottawa, Canada

**Keywords:** Sedentary behaviour, Screen time, Guidelines, Children and youth, School, Child health, Education

## Abstract

**Background:**

Existing sedentary behaviour guidelines for children and youth target overall sedentary behaviour and recreational screen time, without any specific recommendations regarding school-related sedentary behaviours (i.e., sedentary behaviours performed during the school day, or within the influence of school). The purpose of this paper is to describe the development of international evidence-based recommendations for school-related sedentary behaviours for children and youth, led by the Sedentary Behaviour Research Network (SBRN).

**Methods:**

A panel of international experts was convened by SBRN in November 2020 to guide the development of these recommendations for children and youth aged ~ 5–18 years. The recommendations were informed by 1) age-relevant existing sedentary behaviour guidelines, 2) published research on the relationship between overall sedentary behaviour and health, 3) a de novo systematic review on the relationship between school-related sedentary behaviours and health and/or academic outcomes, and 4) a de novo environmental scan of the grey literature to identify existing recommendations for school-related sedentary behaviours.

Draft recommendations were presented to the Expert Panel in June 2021. Following thorough discussion and modifications, updated recommendations were distributed for stakeholder feedback from July 9–26. Feedback was received from 148 stakeholders across 23 countries, leading to additional updates to the recommendations. Following further rounds of discussion and updates with the Expert Panel in August and September 2021, consensus was achieved on the final recommendations.

**Results:**

A healthy day includes breaking up extended periods of sedentary behaviour and incorporating different types of movement into homework whenever possible, while limiting sedentary homework. School-related screen time should be meaningful, mentally or physically active, and serve a specific pedagogical purpose that enhances learning. Replacing sedentary learning activities with movement-based learning activities, and replacing screen-based learning activities with non-screen-based learning activities, can further support students’ health and wellbeing.

**Discussion:**

This paper presents the first evidence-based recommendations for school-related sedentary behaviours for children and youth. These recommendations will support the work of parents, caregivers, educators, school system administrators, policy makers, researchers and healthcare providers interested in promoting student health and academic success.

**Supplementary Information:**

The online version contains supplementary material available at 10.1186/s12966-022-01259-3.

## Introduction

Sedentary behaviour is characterized by an energy expenditure ≤1.5 METs while in a sitting, reclining or lying posture [1]. Sedentary behaviours can be screen-based (e.g., TV, computers, smartphones, video games) or non-screen based (e.g., reading a book, paper-based homework, playing board games). The relationships between sedentary behaviours and student health and academic outcomes are complex, and likely differ for specific sedentary behaviours. Sedentary behaviour per se may have a direct impact on metabolic outcomes [2], whereas other impacts may depend on the activites performed while being sedentary. Some non-screen based sedentary behaviours such as reading and doing homework can be beneficially associated with academic achievement [3]. Screen-based sedentary behaviours often demonstrate deleterious associations with a range of health outcomes among school-aged children and youth aged 5–18 years, including body composition, cardiometabolic risk, behavioural conduct, fitness, self-esteem [3, 4] and sleep [5, 6]. However, screen-based devices may also offer opportunities for novel pedagogical approaches and student engagement, as well as increasing access to education for some students, especially during the COVID pandemic [7].

It should also be noted that many common sedentary activities, including the above examples, do not have to be sedentary in nature. These behaviours are only considered to be sedentary when combined with both low energy expenditure and a sitting, reclining or lying posture. For example active video gaming, or paper-based work at a standing desk, are both ways that common sedentary behaviours can be made non-sedentary. When discussing screen-based or non-screen-based sedentary behaviours in this manuscript, we are referring specifically to behaviours that are assumed to meet the above definition for sedentary behaviour with respect to both energy expenditure and posture.

Public health guidelines focusing on sedentary behaviour have been released by multiple countries [8–12], physician groups [13–15] and the World Health Organization (WHO) [16]. Although there is some variation in the specific recommendations, they typically suggest breaking up periods of extended sedentary behaviour and limiting recreational screen time [8–10, 12, 14, 16]. To date, sedentary behaviour guidelines have not provided specific guidance for school-related sedentary behaviours, with most recommendations focusing on overall sedentary behaviour and “recreational” or “entertainment” screen time instead [8].

At present there are no evidence-based recommendations that can guide parents, caregivers, educators, school system administrators, policy makers, researchers and healthcare providers with respect to specific sedentary behaviours occurring during school hours (e.g., classroom, recess) or outside of school hours but within the influence of the school (e.g., homework), collectively referred to herein as “school-related sedentary behaviour”. Evidence suggests that at present the school day is primarily sedentary [17, 18], and may encourage long periods of uninterrupted sedentary behaviour [19]. For example, Harrington et al. [19] reported that children accumulate a higher number of sedentary periods > 20 min in length during the school-day than in the after-school period or on weekends. Screens have also been incorporated into the classroom setting, being used for behavioural management [20, 21], communication with parents [20, 21], promotion of physical activity [20], and to entertain students during snack and lunch breaks [22]. The shift of homework assignments to online tools such as Google Classroom (Google LLC, Menlo Park, USA), increased reliance on video conferencing and distance-learning during the COVID-19 pandemic, and social-distancing requirements for in-person learning have also contributed to increased school-related sedentary behaviour and screen use, which may have deleterious impacts on other health behaviours and indicators [3–6]. Given the above trends, it is likely that screen-based sedentary behaviours will continue to be increasingly integrated into educational-strategies in the future. This highlights the importance of ensuring that screen-based sedentary behaviours are integrated in ways that support learning and positive academic outcomes, while minimizing potential harms. Educators, parents and school administrators regularly reach out to members of the Sedentary Behaviour Research Network (SBRN) with questions on school-related sedentary behaviours, suggesting that this is a topic of relevance to these key stakeholders.

The school day is a relatively structured environment, providing educators and administrators with an opportunity to influence students’ movement behaviours (and thereby their health) in a positive way. It is important to provide guidance to these groups so that they can take informed action to maximize the benefits and minimize the deleterious impacts of school-related sedentary behaviours for children and youth. Given the large amount of sedentary behaviour accumulated at school and the interest in this topic from key stakeholders, it is surprising that there is limited evidence-based guidance, with no national or international recommendations published to date. The lack of evidence-based recommendations precludes the development of policies to promote wholistic student health and academic achievement.

SBRN is an organization that connects researchers and health professionals with an interest in sedentary behaviour. SBRN’s mission includes disseminating research findings related to sedentary behaviour to both the academic community and the general public. As noted above, SBRN members receive frequent questions on the topic of school-related sedentary behaviour from teachers, school administrators and policy makers. As a result of these discussions, in October 2020 SBRN began a process to develop evidence-based recommendations for school-related sedentary behaviours. The purpose of this report is to describe the process that was used to develop these International School-Related Sedentary Behaviour Recommendations for Children and Youth.

## Methods

**Fig. 1:**
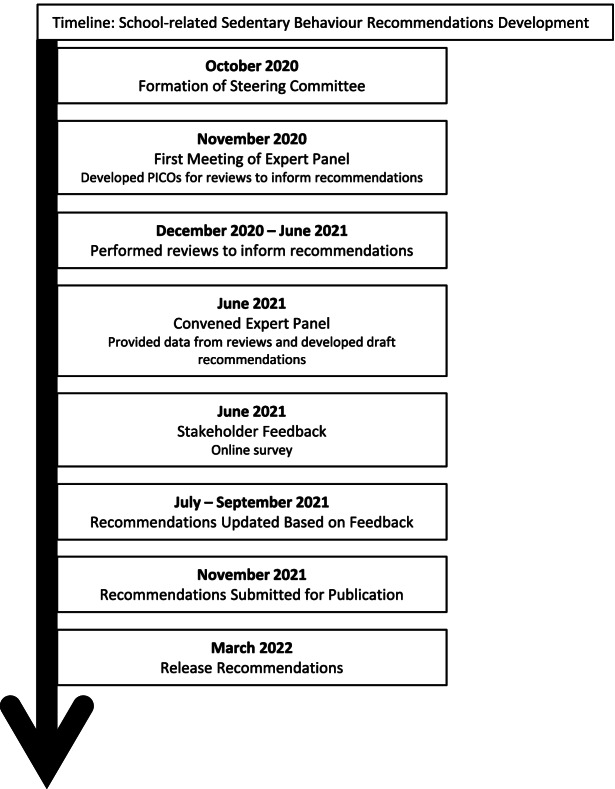
Timeline of School-Related Sedentary Behaviour Recommendations Development

These recommendations were developed using the framework outlined by Tremblay and Haskell [23], which has been used to guide previous movement behaviour guidelines for this age group [12] (Fig. 1). This process began in October 2020 with the establishment of the Steering Committee (TJS, MST, ID, SR, NK), which met regularly throughout the development process. The Steering Committee invited experts from relevant disciplines including research, education, policy, and medicine to form an international Expert Panel (Supplemental File S1). The Expert Panel initially consisted of 22 members; 1 member subsequently asked to be removed from the Panel due to lack of time to attend meetings or provide input. All meetings involving the Expert Panel took place via Zoom (Zoom Video Communications, San Jose, USA). The Expert Panel met for the first time on November 12, 2020 to introduce members of the panel, provide an overview of SBRN and the current project, and obtain feedback on the reviews that were proposed to inform the recommendations [7].

### Evidence used to inform the recommendations

These recommendations were informed by 4 key sources of evidence.

1) Existing age-relevant national and international sedentary behaviour guidelines. These were identified via a recent systematic scoping review on this topic by Parrish et al. [8], which included recommendations published prior to March, 2019. We also included recommendations published by the Canadian Paediatric Society [14], American Academy of Pediatrics [24] and Canadian Association of Optometrists/Canadian Ophthalmological Society [15].

2) A systematic review on the relationship between sedentary behaviour and health among school aged children and youth by Carson et al. [3], which included studies published up until December of 2014. This review was used to inform sedentary behaviour guidelines in Canada, New Zealand and Australia [12, 25, 26].

3) A de novo systematic review on the relationship between school-related sedentary behaviours and health and/or academic outcomes [7], which included studies published up until December of 2020. The search used in this review was developed based on the terms and strategies used in the above-mentioned review by Carson et al. [3], updated to reflect changes in digital technology and to focus on school-related sedentary behaviours.

4) A de novo environmental scan of the grey literature to identify existing recommendations for school-related sedentary behaviours. Details on the methodology of the environmental scan are included in Supplemental Files S2 and S3, with results included below. Briefly, the environmental scan used four search approaches performed in March–June of 2021 (i.e., grey literature databases, advanced Google searches, targeted web-based searches, and content expert consultation) to identify records that outlined evidence-based school-related sedentary behaviour and screen time guidelines/recommendations for schoolchildren. These strategies were loosely adapted from those used in a systematic review of grey literature on guidelines for school-based breakfast programs in Canada [27]. To be eligible for inclusion, records had to include description of evidence-based guidelines/recommendations for the amount, duration, patterns and types of school-related sedentary behaviours for school-aged children. Recommendations could be released by any group (e.g., government, expert groups, non-governmental organizations) at the provincial/state level or above. Documents written in English or those that could be translated into English using Google Translate were eligible for inclusion. Both published empirical reports and grey literature were eligible for inclusion. Quality of included recommendations was assessed using a modified version of the Authority, Accuracy, Coverage, Objectivity, Date, Significance (AACODS) checklist [28, 29] (Supplemental File S4). Each checklist question had three response options; yes, no, and unsure. If the majority of questions were scored yes, then the criterion was given a point. For the final score, the points were added up across criteria, with a maximum of 6 points. A score of ≤2 was categorized as low quality, 3–4 as moderate quality, and ≥ 5 as high quality.

### Drafting of recommendations

The evidence outlined above was presented to the Expert Panel on June 16, 2021. At this meeting, a preliminary draft of the recommendations, developed by the Steering Committee, was presented to the Expert Panel for discussion and feedback. Following thorough and engaged discussion, the recommendations were updated and then distributed to the Expert Panel for additional feedback prior to stakeholder consultations.

### Stakeholder consultations and subsequent revisions

Once draft recommendations were developed by the Expert Panel, a survey was created to obtain stakeholder feedback on the content and wording of the recommendations (Supplemental Files S5 and S6). This survey was based on a similar survey used in the development of Canadian 24-Hour Movement Guidelines [30, 31]. Stakeholders were considered anyone with a personal (e.g., parents, students) or professional (e.g., educators, healthcare providers, policy makers, researchers) interest in sedentary behaviour and/or student health. This survey was considered program evaluation and was provided with an exemption waiver from the University of Prince Edward Island and Children’s Hospital of Eastern Ontario Research Ethics Boards. Survey responses were collected via Google Forms (Google LLC, Menlo Park, USA) from July 9–26, 2021, using a snowball methodology. The survey was disseminated to key stakeholder groups identified by members of the Expert Panel, as well as within the professional networks of Expert Panelists. The survey was also disseminated to all SBRN members via email newsletter. All respondents to the survey were then asked to distribute within their professional networks, to maximize reach. Data were collated and analyzed using Microsoft Excel (Microsoft Corporation, Redmond, USA).

### Revisions

All stakeholder comments were carefully considered by the Steering Committee, leading to several revisions to the recommendations. These updated recommendations and the survey results were distributed again to the Expert Panel via email for comments on August 6, 2021. Based on these comments a final draft was distributed on September 17, 2021, at which point consensus was reached from all members of the Expert Panel.

## Results

### Overall sedentary behaviour and student health

Evidence on this topic was obtained from the systematic review by Carson et al. [3], which included 235 studies with more than 1,650,000 participants aged 5–17 years across 71 countries. They concluded that high durations and/or frequencies of screen-based sedentary behaviours were unfavourably associated with body composition, clustered cardiometabolic risk, fitness, behavioural conduct/pro-social behaviour, and self-esteem. In contrast, higher durations of homework and reading were associated with better academic achievement. It was not assessed whether reading and homework were done using screens or paper-based media. Across all outcomes, the authors identified the quality of evidence as ranging from very low to moderate.

### School-related sedentary behaviour and student health

Evidence on this topic came from the de novo review completed by Kuzik et al. [7]. The systematic review consisted of 116 studies, including over 1.3 million participants and over 1000 extracted associations between school-related sedentary behaviours and a health outcome. The majority of studies collected data in Europe, Asia, and North America (collectively 96%) and in high income countries (71%). Over half of the studies were cross-sectional study designs, and homework was the most frequently observed sedentary behaviour exposure.

Findings from this review suggested more school-related sedentary behaviours were unfavourable for children’s other movement behaviours (i.e., sleep, physical activity, sedentary behaviour outside of school) but favourable for cognitive and social-emotional indicators. More homework was the exposure most frequently observed as favourable for cognitive and social-emotional indicators. However, the benefits of homework were rarely seen in primary school-aged children, and dose-response relationships indicated high levels of homework were unfavourable. Active lessons were the most beneficial exposure for displacing school-related sedentary behaviours, leading to benefits to children’s health and well-being. Quality of evidence across study designs was most frequently rated as very low.

### Existing sedentary behaviour recommendations

Existing national sedentary behaviour recommendations were identified via the systematic review by Parrish et al. [8]. They reported that numerous countries recommend breaking up periods of extended sedentary behaviour and limiting screen time, although the specific thresholds for each varied across countries. Existing recommendations for digital media use from physician groups in Canada and the United States were also examined [13–15]. Recommendations from pediatric physicians in both Canada and the United States focused primarily on the quality of screen-based media usage to promote cognitive, psychosocial and physical health while minimizing risks [13, 14] while a position statement from the Canadian Association of Optometrists/Canadian Ophthalmological Society recommended limiting recreational screen time to 1–2 h/day, with breaks involving whole-body physical activity every 30–60 min [15].

The de novo environmental scan identified seven publications meeting all eligibility criteria for school-related sedentary behaviour recommendations (Supplemental Files S7 and S8). Four of the seven guidelines or recommendations were from government sources (e.g., Department of Education) at the provincial/state level [32–35]. Two of the guidelines or recommendations were published at the national level by government organizations [36, 37]. One set of recommendations was published in a WHO’s Health Behaviour in School-Aged Children Fact sheet [38]. The guidelines and/or recommendations were all released between 2017 and 2021 and stemmed from the United States (*n* = 2 [32, 35]), India (*n* = 1 [36]), the Philippines (n = 1 [37]), Canada (n = 1 [33]), Australia (n = 1 [34]), and the WHO (*n* = 1 [38]). Based on the modified AACODS checklist, all 7 recommendations were categorized as high quality.

One of the identified documents [32] included general recommendations for digital device use, including: “plan for purposeful and strategic integration of digital resources that support and enhance teaching and learning”, “design learning opportunities that include and promote active engagement”, “consider age and developmental level of students and recognize the importance of time limits”, and that “use of digital devices with younger students be limited”. Two recommendations [36, 37] suggested time limits for online classes/digital education, ranging from 30 min to 1 h for pre-primary/kindergarten students to 3 to 4 h for Grade 9–12 students [36, 37]. Four recommendations [32–34, 38] included suggestions for limiting/reducing sedentary behaviour and breaking up prolonged periods of sedentary time and/or screen time during the school day. For example:

*To enhance their well-being and achievement, all students should strive to achieve high levels of physical activity and limit sedentary behaviour every day. To support them in reaching this goal, educators may want to consider breaking up longer periods of sedentary time during the school day by building movement opportunities into instructional time* [33]*.*

Another recommendation suggested: “limit time on devices – 10 to 20 minutes is recommended. Remind students to take eye and stretch breaks” [32].

Finally, one document addressed digital device use for different age ranges and developmental levels, the amount of time students spend on devices both in the classroom and at home, the appropriate frequency of breaks from screen time, and physical positioning with regard to ergonomics and posture [35]. Example recommendations included, “use digital devices as a classroom tool to promote critical thinking, creativity, communication, etc.” and “take breaks and include physical movement [take a one- or two-minute break every 15 to 20 minutes, or a five-minute break every hour]” [35].

### Stakeholder consultations

As noted above, feedback on draft recommendations was solicited via online survey. The stakeholder survey was completed by 148 individuals from 23 countries, with a mean age of 42 years (Range: 16–69 years). A full summary of the survey results and respondent demographics are available in Supplemental File S6. Stakeholders represented several key groups including researchers (45.3%), educators (35.8%), healthcare workers (15.5%), government (13.5%), parents (10.1%), and post-secondary students (7.4%) (several individuals belonged to multiple groups). Unless otherwise specified, results are presented for all respondents combined.

Respondents were asked to rate their level of agreement/disagreement with each component of the recommendations (i.e. “I _____________ with how the recommendations are stated.”), as well as rate their agreement/disagreement that the component was clearly stated (i.e. “The recommendations are clearly stated”). When asked about their level of agreement with the title, preamble, and glossary, the majority of respondents “strongly agreed” or “somewhat agreed” that the title was clearly stated (86.3%) and with how it was stated (80%), that the preamble was clearly stated (89.2%) and with how it was stated (83%), and that the glossary was clearly stated (88.5%) and with how it was stated (88.4%).

Regarding the agreement with draft recommendations/statements, the majority of respondents “strongly agreed” or “somewhat agreed” that the recommendations were clearly stated (90.5%) and with how they were stated (84.4%). The majority of survey respondents felt that the recommendations were appropriately specific (77.2%) and realistic and achievable for educators (65.3%), students (63.5%), school administrators (63.4%), and parents (62.2%). Among educators and parents, 78.0 and 92.9% respectively agreed that the recommendations were realistic and achievable for members of their group. Of the respondents, 65.8 and 66.0% indicated that the recommendations would be useful in their professional and personal life, respectively. Among healthcare workers and teachers respectively, 69.6 and 80.0% agreed that the recommendations would be useful in their professional life.

Regarding the importance, use, and costs/benefits of the recommendations, the majority of survey respondents considered them “very important” or “somewhat important” (78.2%) and “very relevant” or “somewhat relevant” (78.2%) to their professional work. Among educators, 90.0% indicated that the recommendations would be somewhat or very relevant to their professional work. The majority of respondents also reported that they would use these recommendations in their professional work “always” or “very often” (60%) and find these recommendations “very easy” or “easy” (57.6%) to use. Of the respondents, 43.2% reported they “strongly agree” or “somewhat agree” that the costs (e.g., time, financial, opportunity) for them or their organization to implement these recommendations would likely be small or negligible compared to not implementing them, while 12.3% disagreed. The majority of respondents (67.8%) felt that the benefits of using these recommendations were likely to outweigh the costs in their professional work, and that following these recommendations is likely to benefit students regardless of gender, race, ethnicity, nationality, or socioeconomic status, as indicated by 87.0% of respondents.

In terms of the level of agreement with proposed implementation strategies, the majority of respondents “strongly agreed” or “somewhat agreed” that the implementation strategies were clearly stated (93.1%) and with how they were stated (89%). The respondents considered the proposed implementation strategies to be realistic and achievable for educators (75.2%), healthcare providers (75%), school administrators (73.8%), students (72.8%), and parents (70%). Similar findings were observed when respondents were asked about their own group; 84% of educators, 73.9% of healthcare providers and 78.6% of parents somewhat or strongly agreed that the implementation strategies were realistic and achievable for members of the same group. Of the survey respondents, 76.2 and 75.3% indicated that the proposed implementation strategies would be useful in their professional and personal life, respectively.

When asked to provide an example of how they would apply these recommendations in their own work, stakeholders provided a range of examples. Educators indicated that they would implement them within their own classrooms, or use them to advocate for change within their school or department. Researchers stated that they would assess the number of students meeting these recommendations in population surveillance research, or use them as targets in intervention-based studies. Healthcare providers indicated that they would share the recommendations with parents and teachers, and use them to advocate for policy changes within schools.

### Revisions based on stakeholder feedback

A number of small changes were made to the draft recommendations based on the stakeholder feedback. This included removing “SBRN” from the title, making the glossary definitions more appropriate to a lay-audience, providing additional details related to the duration and intensity of movement breaks, and making device-breaks consistent with movement breaks. The revised recommendations were circulated to the Expert Panel for an additional round of feedback and revision. Final recommendations were circulated to the Expert Panel for approval on September 17, at which point consensus was reached from all panelists.

### Recommendations

**Fig. 2:**
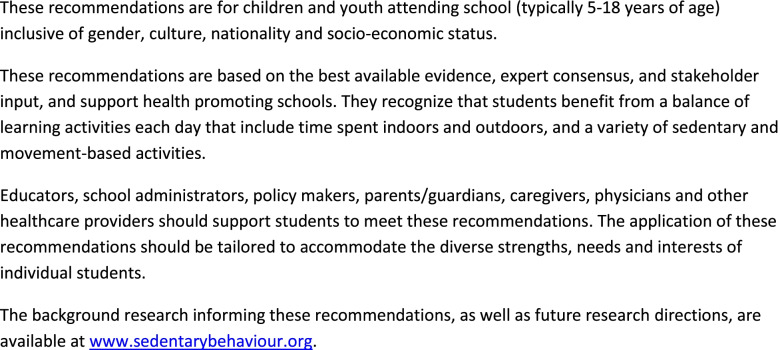
Pre-amble to School-Related Sedentary Behaviour Recommendations

**Fig. 3:**
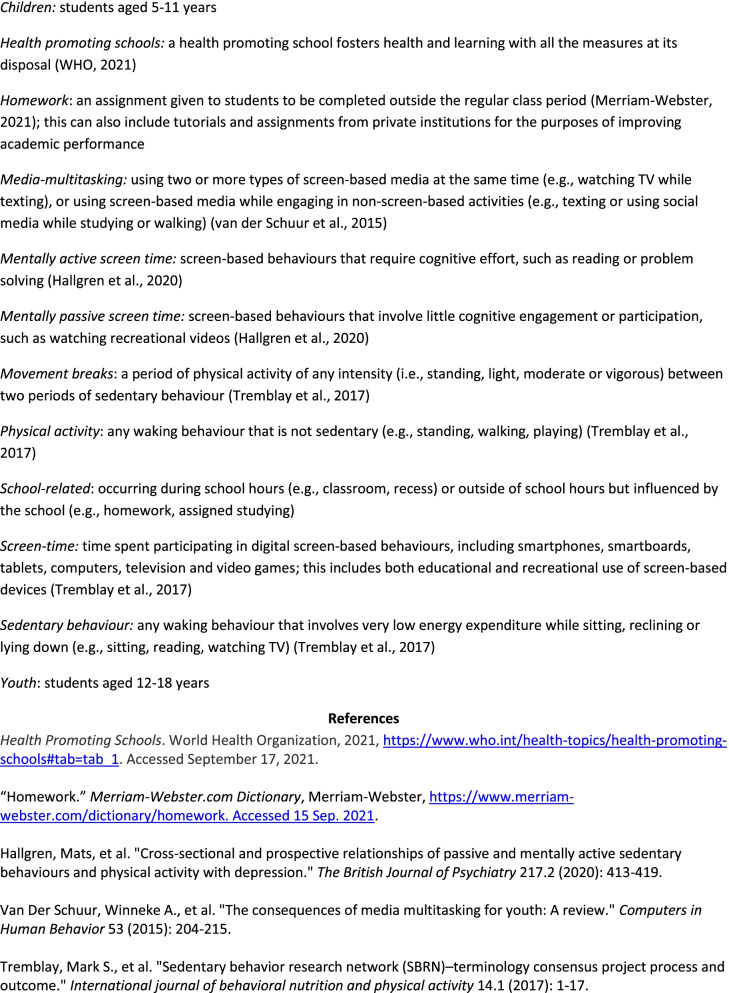
Glossary of Terms Included in School-Related Sedentary Behaviour Recommendations

**Fig. 4:**
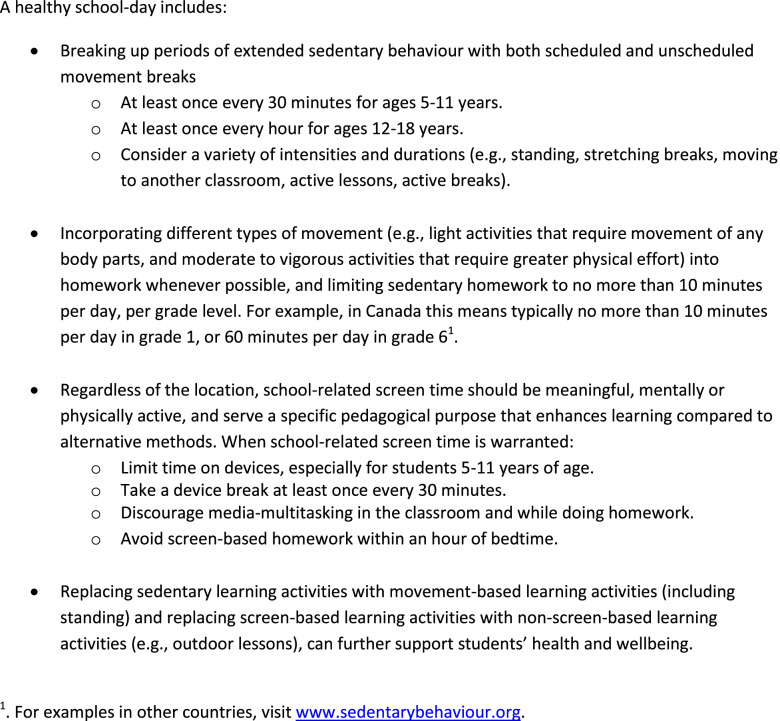
School-Related Sedentary Behaviour Recommendations for Children and Youth

**Fig. 5:**
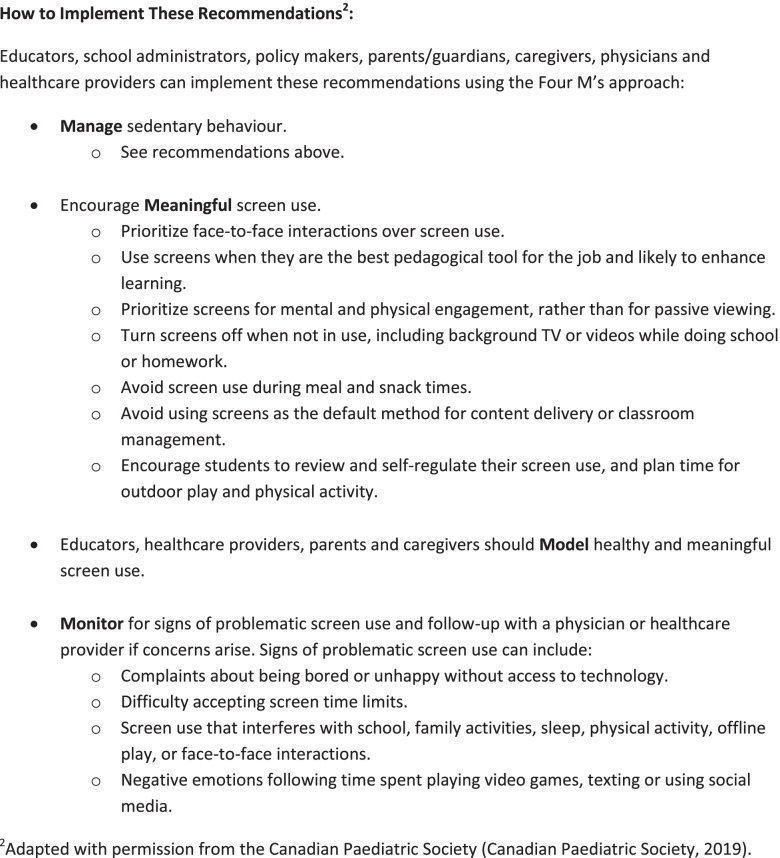
Implementation Strategies for School-Related Sedentary Behaviour Recommendations

The final pre-amble, glossary, recommendations and implementation strategies are shown in Figs. 2, 3, 4 and 5. The recommendation that students should break up periods of extended sedentary behaviour (Recommendation 1) was based on similar recommendations in the national physical activity guidelines of several nations [8] and in the school-related recommendations identified in our environmental scan [32–34, 38], as well as the findings of Kuzik et al. [7], which concluded that active lessons were associated with favourable health outcomes for students. More frequent breaks were suggested for students aged 5–11 years based on similar recommendations in national physical activity recommendations [8], as well as the recognition that there are distinct developmental differences between early childhood and adolescence.

The recommendation that movement be incorporated into homework (Recommendation 2) was based on the wealth of evidence highlighting the benefits of physical activity for children and youth [39]. The recommendation that sedentary homework be limited to no more than 10 min per grade, per day, was based on the “10-min rule” endorsed by the National Education Association and National Parent Teacher Association of the United States [40]. It also recognises that students need sufficient free time to engage in other activities that promote healthy physical and social development. Although the English-language recommendations presented in this paper include an example based on the grade system used in English-speaking North America, when translating the recommendations into additional languages an example from a relevant country will be provided for local context.

Recommendation 3 highlights ways that students can experience the developmental and pedagogical benefits of screen use, while minimizing the harms. This recommendation was informed by evidence suggesting that screen time shows unfavourable associations with numerous health outcomes among children and youth, and public health recommendations to limit recreational screen time in this age group [3, 8]. It also recognizes that purposeful screen time developed as a learning tool is associated with learning benefits, which may be especially relevant in developing nations [7]. It encourages breaking up screen use at least once every 30 min, which is in line with previous recommendations to minimize musculoskeletal and vision problems associated with uninterrupted screen use [15, 35]. These recommendations discourage media multitasking, which has been shown to have deleterious associations with learning outcomes [41]. It is worth noting that our definition of media multitasking focuses on screen use, but does not include listening to music, as current evidence on the impact of listening to music while studying remains unclear [42]. Finally, the recommendation to encourage students to avoid screen-based homework within 1 h of bedtime was based on similar recommendations by the Canadian Paediatric Society, American Academy of Pediatrics, Canadian Association of Optometrists/Canadian Ophthalmological Society, and Virginia Department of Education [13–15, 35], as well as evidence linking screen use with reduced sleep duration and quality [5, 43].

Recommendation 4 recognizes that in general, incorporating movement into the school day and limiting overall screen use are likely to positively impact student health outcomes [3, 39], and are unlikely to be associated with harms.

## Discussion

In this paper we outline the process used to develop evidence-based recommendations for school-related sedentary behaviours. They were developed following an established process to ensure rigor and transparency, and considered evidence collected through comprehensive reviews, the input of an international panel of experts, and extensive stakeholder feedback. These recommendations recognise that sedentary behaviours are a normal and appropriate part of the school day, and provide ways that students, parents, educators and other stakeholders can obtain the benefits of sedentary behaviour, while minimising any negative impacts on health or academic outcomes. The overall quality of available evidence used to inform these recommendations was most frequently identified as very low, indicating a reliance on observational studies at risk of one or more forms of serious bias. However, the benefits of adopting these new recommendations are likely to outweigh the risk of harm.

The development of these recommendations identified a number of important research gaps of relevance to school-related sedentary behaviours. As outlined in Kuzik et al. [7], this includes more high-quality experimental and longitudinal study designs, more research conducted in low-middle-income countries, and a continued reflection on how research is contributing to inclusive and equitable quality education for all [7]. The current recommendations focus on school-aged children and youth ~ 5–18 years of age. There may be a need for future recommendations focusing on education or care-related sedentary behaviours for other age groups, including infants, toddlers and pre-school aged children, as well as adults learning in post-secondary environments.

As noted elsewhere, public-health recommendations often drive research, leading to higher quality evidence that can be used to refine future recommendations [44]. The majority of respondents felt that these recommendations would be feasible to implement. This is similar to previous recommendations for overall physical activity and sedentary behaviour in other age groups [30, 45], despite low levels of adherence in the general population [46, 47]. Differences between perceived feasibility to implement recommendations and actual implementation could result from stakeholder samples lacking population generalizability, or could be part of the broader intention-behaviour gap observed in other sedentary behaviour literature [48]. Therefore, future research is needed to further investigate the perceived and actual feasibility of these recommendations, and their impact on student health, well-being and learning. Of note, several researchers (18.6%) who completed the stakeholder survey indicated that they would assess adherence to these recommendations in population surveillance work and/or use the recommendations as targets in intervention studies, which will help to address the above issues. This work is also likely to improve our understanding of the relationship between school-related sedentary behaviours and both health and academic outcomes. Challenges to these recommendations are encouraged and revisions or updates based on emerging evidence are warranted.

These recommendations support ongoing efforts to promote student health, such as the WHO’s Health Promoting Schools [49] and the Canadian Healthy School Standards [50]. They also recognise the importance of understanding both the positive and negative impacts that sedentary behaviours can have on health and academic outcomes, in order to promote healthy physical and cognitive development. These recommendations are relevant to all school-aged students, although they may need to be modified to meet the needs of individual students. Sedentary behaviour involves both a postural component (sitting, reclining, lying) and an energy expenditure component (≤1.5 METs) [1]. Therefore students can break up or limit their sedentary behaviour by engaging in movement that increases their energy expenditure above resting while seated or standing, based on their needs and preferences. The recommendations use the term “movement” rather than “physical activity” to highlight that sedentary behaviour can be reduced or broken up by activity of any intensity, and does not require that students engage in structured physical activity. In order to maximize their utility and impact, the recommendations require translation into additional languages, along with examples appropriate to locations where each language is spoken.

### Strengths and weaknesses

These recommendations were informed by the best available evidence and developed in consultation with an international Expert Panel, in response to an evidence gap identified by key stakeholders. Draft recommendations received input from key stakeholders in 23 countries, representing relevant key stakeholders including parents, caregivers, educators, school system administrators, policy makers, researchers and healthcare providers. We did not specifically update recent reviews performed by Parrish [8] and Carson [3]. However the de novo review by Kuzik et al. [7] was based on the search strategy used by Carson et al. [3], while recent sedentary behaviour recommendations known to the Expert Panel were considered in addition to those identified by Parrish et al. [8]. These recommendations are limited by the quality of available evidence, which was very low. Sedentary behaviours included in the review by Kuzik et al. [7] were typically self-reported, and both screen time and homework were assumed to be sedentary, but this was not confirmed via device-based measures. Further, the available evidence focused primarily on able-bodied students with typical development. However, we believe that the benefits of implementing these recommendations are likely to outweigh any potential harms regardless of student ability. To ensure that these recommendations are relevant to all students, the pre-amble also notes that they may need to be adapted to meet the diverse strengths, needs, and interests of individual students. These recommendations fill a void identified by educators struggling to cope with high levels of student sedentary behaviour, screen ubiquity and concerns that increases in sedentary behaviour and screen time due to COVID-19 may become regularized once the pandemic ends. These recommendations were developed according to an accepted and recognized process [23]. Within the financial and practical constraints of the current project, we were not able to follow all components of the Appraisal of Guidelines, Research and Evaluation (AGREE II) framework [51], in particular, having the guideline and process externally reviewed prior to publication or committing to a timeline for future updates of the recommendations. However, we have nonetheless followed a robust and transparent approach involving key stakeholders and the collection of the best available evidence on school-related sedentary behaviours and both health and academic outcomes. Given the important role that schools can play in the promotion of healthy behaviors, we encourage national and international public health agencies to consider inclusion of specific recommendations related to the school environment in future sedentary behaviour guidelines.

### Conclusion

The International Recommendations for School-Related Sedentary Behaviours for Children and Youth presented in this paper provide guidance to parents, educators, policymakers, researchers and healthcare providers. They were developed by a panel of international experts and informed by the best available evidence and stakeholder consultation. These recommendations will be useful in supporting the physical and mental health, well-being and academic success of school-age children and youth.

## Supplementary Information

**Additional file 1: S1.** Expert Panel Membership.

**Additional file 2: S2.** Environmental Scan Methodology.

**Additional file 3: S3.** Advanced Google Searches.

**Additional file 4: S4.** Modified AACODS Checklist.

**Additional file 5: S5.** Stakeholder Questionnaire.

**Additional file 6: S6.** Stakeholder Feedback Responses.

**Additional file 7: S7.** Environmental Scan Flow Diagram.

**Additional file 8: S8.** Characteristics of Included Guidelines.

## Data Availability

Summaries of the data used to inform these recommendations are available in Supplemental Files 6 and 8. Raw data from the stakeholder survey are available from the corresponding author on reasonable request.
