# Supplementary material for: International school-related sedentary behaviour recommendations for children and youth
Source: Int J Behav Nutr Phys Act. 2022 Apr 5;19:39. doi: 10.1186/s12966-022-01259-3 (PMC8979784; doi:10.1186/s12966-022-01259-3)
Supplement: Supplementary file 1 — Additional file 1: S1. Expert Panel Membership. [file 12966_2022_1259_MOESM1_ESM.docx]

Table 1. Expert Panel Membership

| Panel Members | Affiliation | Role | Conflict of interest declaration |
| --- | --- | --- | --- |
| Stacey Bélanger | Digital Health Task Force, Canadian Paediatric Society, Ottawa, Canada | Canadian Paediatric Society Representative, Pediatrician, Systematic Review Author | None |
| Kara Brisson-Boivin | Mediasmarts, Ottawa, Canada | MediaSmarts Director of Research, Systematic Review Author | None |
| Valerie Carson | Faculty of Kinesiology, Sport, and Recreation, University of Alberta, Edmonton, Canada | Researcher, Systematic Review Author | None |
| Bruno Gonçalves Galdino da Costa | School of Physical and Health Education, Nipissing University, North Bay, Canada | Researcher, Systematic Review Author | None |
| Melanie Davis | Physical and Health Education Canada, Ottawa, Canada | Physical and Health Education Canada Representative, Systematic Review Author | None |
| Iryna Demchenko | Healthy Active Living and Obesity Research Group, Children’s Hospital of Eastern Ontario Research Institute, Ottawa, Canada | Steering Committee Member | None |
| Susan Hornby | Pan-Canadian Joint Consortium for School Health Secretariat, Summerside, Canada | Joint Consortium for School Health Representative, Policy Maker, Systematic Review Author | None |
| Wendy Yajun Huang | Department of Sport, Physical Education and Health, Hong Kong Baptist University, Hong Kong, China | Researcher, Systematic Review Author | None |
| Nicholas Kuzik | Healthy Active Living and Obesity Research Group, Children’s Hospital of Eastern Ontario Research Institute, Ottawa, Canada | Steering Committee Member, Researcher, Systematic Review Lead Author | None |
| Barbi Law | School of Physical and Health Education, Nipissing University, North Bay, Canada | Researcher, Systematic Review Author | Dr Law reports a research partnership with Active for Life, and research funding from the Canadian Institutes of Health Research to study Canadian 24-Hour Movement Guidelines adherence. |
| Chris Markham | Ontario Physical and Health Education Association, Ottawa, Canada | Ontario Physical and Health Education Association Representative | None |
| Michelle Ponti | Digital Health Task Force, Canadian Paediatric Society, Ottawa, Canada | Canadian Paediatric Society Representative, Pediatrician | None |
| Maribeth Rogers Neale | Department of Education and Lifelong Learning, Government of Prince Edward Island, Summerside, Canada | Prince Edward Island Department of Education and Lifelong Learning Representative, Educator | None |
| Scott Rollo | Healthy Active Living and Obesity Research Group, Children’s Hospital of Eastern Ontario Research Institute, Ottawa, Canada | Steering Committee Member, Researcher, Environmental Scan Lead Author, Systematic Review Author | None |
| Jo Salmon | Institute for Physical Activity and Nutrition, Deakin University, Geelong, Australia | Researcher, Systematic Review Author | Dr Salmon reports research grants from the National health and Medical Research Council of Australia to study the efficacy, effectiveness and implementation of TransformUs program in primary schools. She reports that her spouse has developed a height-adjustable desk business for schools, although this is not currently operational. |
| Travis Saunders | Department of Applied Human Sciences, University of Prince Edward Island, Charlottetown, Can | Steering Committee Chair, Researcher, Systematic Review Author | Dr Saunders reports research funding from the Levesque Foundation and the Public Health Agency of Canada to study the impacts of sedentary behaviour, assisted the Canadian Society for Exercise Physiology in developing materials related to sedentary behaviour measurement and interventions. He has received honoraria for public talks on the health impact of sedentary behaviour, and was the sedentary behaviour content lead for Canada’s 24-Hour Movement Guidelines for Adults 18-64 years and 65+ years. |
| Jennifer R Tomasone | School of Kinesiology and Health Studies, Queen’s University, Kingston, Ontario, Canada | Researcher, Systematic Review Author | Dr Tomasone was the Knowledge Translation Lead for the Canadian 24-Hour Movement Guidelines for Adults 18-64 years and 65+ years. |
| Mark Tremblay | Healthy Active Living and Obesity Research Group, Children’s Hospital of Eastern Ontario Research Institute, Ottawa, Canada | Sedentary Behaviour Research Network Representative, Steering Committee Member, Researcher, Systematic Review Author | Dr Tremblay reports publishing papers related to sedentary behaviour, and is the volunteer Chair of the Sedentary Behaviour Research Network. |
| Antonius J Van Rooij | Department of Youth, Trimbos Institute, Utrecht, The Netherlands | Researcher | Dr. Van Rooij reports research funding from Dutch governmental departments and Dutch scientific grant agencies in various projects to study and address the negative and positive effects of games, gambling, and media use. Trimbos Institute occasionally receives honoraria for speaking engagements by Dr. Van Rooij on these subjects. |
| Lucy-Joy Wachira | Physical Education, Exercise and Sports Science, Kenyatta University, Nairobi, Kenya | Researcher, Systematic Review Author | None |
| Katrien Wijndaele | MRC Epidemiology Unit, University of Cambridge, Cambridge, UK | Researcher, Systematic Review Author | Dr Wijndaele is an Associate Editor at International Journal of Behavioural Nutrition and Physical Activity, past co-chair of the International Society for Physical Activity and Health Sedentary Behaviour Council, and reports funding from the UK Medical Research Council (MC_UU_12015/3). |
