# Supplementary material for: International school-related sedentary behaviour recommendations for children and youth
Source: Int J Behav Nutr Phys Act. 2022 Apr 5;19:39. doi: 10.1186/s12966-022-01259-3 (PMC8979784; doi:10.1186/s12966-022-01259-3)
Supplement: Supplementary file 2 — Additional file 2: S2. Environmental Scan Methodology. [file 12966_2022_1259_MOESM2_ESM.docx]

Supplemental File S1. Environmental Scan Methodology

**Information sources and search strategy.** Four different search approaches (i.e., grey literature databases, advanced Google searches, targeted web-based searches, and content expert consultation) were used to identify records (e.g., empirical studies, organizational reports, website pages, guideline messages) that outline evidence-based school-related sedentary behaviour and screen time guidelines/recommendations for schoolchildren. These strategies were adapted from those used in a systematic review of grey literature on guidelines for school-based breakfast programs in Canada (1).

The first search strategy involved searching grey literature databases relevant to the subject of the review. A search was conducted on May 24, 2021 and included six databases: Canadian Research Index (ProQuest, Micromedia), the Canadian Public Documents Collection, TRIP Medical Database, OpenGrey, ECRI Guidelines Trust, and National Institute for Health and Care Excellence (UK). The search strategy included the following five groups of terms: (1) sedentary behaviour (e.g., sedentary, stationary, sitting); (2) screen time; (3) guidelines (e.g., guidelines, recommendations, standards, frameworks, best practices); (4) school (e.g., school, education, classroom, student); and (5) children (e.g., child, youth, adolescent). Because the databases had a wide variance in search functionalities and filters available for retrieving results, the specific search strategy and terms used were adapted to fit the specific database used. The results of the database searches were exported to an Excel spreadsheet and the titles of all records retrieved were reviewed by one author (SR). Titles that appeared relevant were highlighted in Excel and retained for further screening.

The second search strategy involved conducting Google searches for documents published on the Internet using the Google Advanced Search engine. Ten unique search strategies were applied, as shown in Supplemental File S2. The first ten pages of each search’s hits (representing 100 results) were reviewed, using the title and short text underneath. This number of pages was chosen to capture many of the most relevant hits while still being a feasible amount to screen. Potentially relevant records were ‘bookmarked’ in the web browser used at the time of searching (Google Chrome) and later entered into an Excel spreadsheet. Each bookmarked homepage was filed into a sub-folder that was named after the specific search strategy by which it was identified, which enabled the reviewers to access the bookmarked websites via the browser’s Bookmark Manager, see which websites were identified through which search terms, and prevent the same record from being identified repeatedly throughout this search strategy. This feature allowed us to easily track new records identified through each search, since the URL of previously bookmarked pages were starred when viewing the page. Titles that were identified as potentially relevant were retained for further screening. For each search strategy, the search terms and the number of results retrieved and/or screened were recorded.

The third search strategy involved browsing targeted websites of relevant organizations and agencies (e.g., government, health, education, non-government, universities, research centres, etc.) (n = 50), which were identified and selected by the steering committee, as well as soliciting recommendations from the guideline development expert panel. The targeted web searches took place on June 14, 2021. Each of the relevant websites were searched using the websites’ search bar and/or ‘hand-searched’ for potentially relevant documents.

The fourth search strategy involved contacting content experts and SBRN members to identify any existing guidelines for possible inclusion in the review. Specifically, each of the members of the guideline development expert panel (i.e., researchers, teachers, policy makers, and pediatricians) were contacted by email on March 17, 2021. Further, an e-blast was sent to all SBRN members (n = 1693) on the same date; 498 (29.4%) recipients across 10 countries opened the email. The email message briefly explained the aims of the project and requested that the recipient identify or send any potentially relevant documents or sources and/or forward the message to colleagues who could potentially provide assistance. All items identified by way of the content expert consultation were considered and proceeded for further screening.

**Eligibility assessment and study selection.** The title and source organization of documents identified and deemed relevant from the four search approaches were entered into an Excel sheet, and duplicates were excluded. The abstracts, executive summaries, and/or tables of contents (whichever were available) of items were reviewed for relevance against the eligibility criteria by one author (SR). Next, the full-text of all items that moved to the second stage of screening was reviewed. When it was unclear whether or not an item met the eligibility criteria during screening, the reviewer erred on the side of caution and the item continued for further screening. All items that remained following full-text screening were included in the review.

**Data collection process and synthesis of results.** Following full review of each included publication, data were extracted by SR pertaining to the country, title of guidelines, issuing authority/source organization, date of release, who they were developed by, intended audience, goal/objectives of document, sources of evidence/resources cited, and recommendations for school-related sedentary behaviour and/or screen time. Only data that were relevant to recommendations focusing on sedentary behaviour or screen time performed within the school day, as well as sedentary behaviours assigned by the school (e.g., homework) were extracted, consistent with the a priori objectives of the review.

A modified version of the AACODS checklist (2) was used to evaluate and critically appraise the quality of each guideline meeting the above criteria and included in the environmental scan. Briefly, the checklist included six assessment criteria, consisting of one or more questions each, including Authority (reputable, authority in the field), Accuracy (aim/brief, references, representative of field), Coverage (limits stated), Objectivity (clear standpoint, balanced), Date (clear date, recent date), and Significance (meaningful, unique, impactful). Each question had three response options; yes, no, and unsure. If the majority of questions were scored yes, then the criterion was given a point. For the final score, the points were added up across criteria, and a score of ≤2 was categorized as low quality, 3-4 as moderate quality, and ≥5 as high quality.

**References**

1. Godin K, Stapleton J, Kirkpatrick SI, Hanning RM, Leatherdale ST. Applying systematic review search methods to the grey literature: a case study examining guidelines for school-based breakfast programs in Canada. Syst Rev. 2015 Oct 22;4:138.

2. Tyndall J. AACODS Checklist [Internet]. 2010 [cited 2021 Oct 27]. Available from: https://canberra.libguides.com/c.php?g=599348&p=4148869
