# Supplementary material for: International school-related sedentary behaviour recommendations for children and youth
Source: Int J Behav Nutr Phys Act. 2022 Apr 5;19:39. doi: 10.1186/s12966-022-01259-3 (PMC8979784; doi:10.1186/s12966-022-01259-3)
Supplement: Supplementary file 3 — Additional file 3: S3. Advanced Google Searches. [file 12966_2022_1259_MOESM3_ESM.docx]

# Supplemental File XX. Search results – Advanced Google search

| **#** | **Search** | **# results** | **# results screened** | **# new potentially relevant records** | **Total # records** |
| --- | --- | --- | --- | --- | --- |
| 1 | sedentary OR screen time AND guidelines AND children AND school | ~ 203,000,000 | 100 | 72 | 72 |
| 2 | sedentary behaviour AND guidelines AND children OR youth | ~ 3,440,000 | 100 | 19 | 91 |
| 3 | screen time AND guidelines AND children OR youth | ~ 335,000,000 | 100 | 15 | 106 |
| 4 | sedentary AND guidelines AND school OR education | ~ 14,000,000 | 100 | 17 | 123 |
| 5 | screen time AND guidelines OR policy AND school OR education | ~ 2,860,000,000 | 100 | 15 | 138 |
| 6 | sedentary OR screen time AND recommendations AND school | ~ 2,800,000,000 | 100 | 1 | 139 |
| 7 | sedentary OR screen time AND recommendations AND children OR youth | ~ 2,380,000,000 | 100 | 0 | 139 |
| 8 | school guidelines AND sedentary OR screen time | ~ 422,000,000 | 100 | 5 | 144 |
| 9 | electronic devices AND guidelines AND school OR education | ~ 191,000,000 | 100 | 19 | 163 |
| 10 | stationary OR sitting AND guidelines OR policy AND school OR education | ~ 1,830,000,000 | 100 | 3 | 166 |
