# Supplementary material for: International school-related sedentary behaviour recommendations for children and youth
Source: Int J Behav Nutr Phys Act. 2022 Apr 5;19:39. doi: 10.1186/s12966-022-01259-3 (PMC8979784; doi:10.1186/s12966-022-01259-3)
Supplement: Supplementary file 4 — Additional file 4: S4. Modified AACODS Checklist. [file 12966_2022_1259_MOESM4_ESM.docx]

| **AACODS** | **Criteria** | **Yes** | **No** | **?** |
| --- | --- | --- | --- | --- |
| **Authority** | Organization or group:  • Is the organization reputable? (e.g. W.H.O)  • Is the organization an authority in the field? |  |  |  |
| **Accuracy** | • Does the item have a clearly stated aim or brief? If so, is this met?  • Supported by authoritative, documented, up-to-date references, or credible sources?  • Is it representative of work in the field? If no, is it a valid counterbalance? |  |  |  |
| **Coverage** | All items have parameters which define their content coverage. These limits might mean that a work refers to a particular population group. A resource could be designed to address a particular health behavior or a specific topic within a health behaviour.  • Are any limits clearly stated? (i.e. for childcare centres, age group, health behaviours) |  |  |  |
| **Objectivity** | It is important to identify bias, particularly if it is unstated or unacknowledged.  • Opinion, or otherwise, is still opinion: is the organization's standpoint clear?  • Does the work seem to be balanced in presentation? |  |  |  |
| **Date** | For the item to inform your research, it needs to have a date that confirms relevance  • Does the item have a clearly stated date related to content? If no date is given, but can be closely ascertained, is there a valid reason for its absence?  No date, no valid reason: ?  •Resource date  <5 years: Yes  5-10 years: ?  >10 years: No  No date: ? |  |  |  |
| **Significance** | This is a value judgment of the item, in the context of the relevant research area  • Is the item meaningful? (this incorporates feasibility, utility and relevance)  • Does it enrich or add something unique to the list of resources? Would the list of resources be lesser without it?  • Does it have impact? (in the sense of influencing the work or behaviour of others) |  |  |  |

**Scoring:**

Each of the 6 categories receives a point if most questions within the category were marked yes.

Final scores:
≤2 points = low quality
3-4 points = moderate quality
≥5 points = high quality

Adapted from: AACODS Checklist. Jessica Tyndall, Flinders University, 2010. Available from: [https://dspace.flinders.edu.au/xmlui/bitstream/handle/2328/3326/AACODS_Checklist.pdf;jsessionid=AB22D6D950708079623DDC5569D5FA45?sequence=4](about:blank)

Adapted in: An Environmental Scan of Existing Canadian Childcare Resources Targeting Improvements in Health Behaviours. Early Childhood Educ J (2021). Carson, V., Predy, M., Hunter, S. et al. <https://doi.org/10.1007/s10643-021-01266-2>
