# Supplementary material for: International school-related sedentary behaviour recommendations for children and youth
Source: Int J Behav Nutr Phys Act. 2022 Apr 5;19:39. doi: 10.1186/s12966-022-01259-3 (PMC8979784; doi:10.1186/s12966-022-01259-3)
Supplement: Supplementary file 5 — Additional file 5: S5. Stakeholder Questionnaire. [file 12966_2022_1259_MOESM5_ESM.pdf]

SBRN School-Related Sedentary Behaviour Recommendations Public Consultation

The Sedentary Behaviour Research Network (SBRN) is the only organization for researchers and health professionals which focuses specifically on the health impact of sedentary behaviour. SBRN's mission is to connect sedentary behaviour researchers and health professionals working in all fields of study, and to disseminate this research to the academic community and to the public at large.

Although pediatric public health guidelines have been developed for sedentary behaviour and recreational screen time, to date there are no similar recommendations focusing on school-related sedentary behaviours. Working with a team of international experts over the past year, SBRN has drafted evidence-based recommendations for school-related sedentary behaviours to fill this important gap.

The purpose of this survey is to gather feedback on these draft recommendations. We expect this survey to take about 20 minutes to complete.

Information in this form is collected anonymously. Summary data from this survey and anonymous quotes may be included in publications and research presentations related to the recommendations, as well as being posted to the SBRN website (www.sedentarybehaviour.org). Participation in this survey is voluntary; you may skip questions or close the survey at any time. If you have any questions or concerns about this project or this survey, please email Travis Saunders (trsaunders@upei.ca). This survey will be available from July 9-25, 2021.

Please note that these are DRAFT recommendations, which will be updated following this consultation process. We encourage you to share this survey within your professional networks. Thank you for your input.

* Required

1. I consent to participating in this survey (if not, please close the survey now). *

   Mark only one oval.

   ◯ I consent.

2. Age (years)

3. Language spoken most frequently at home

4. Gender

Mark only one oval.

- Female
- Male
- Non-binary
- Prefer not to say
- Other:

5. Nationality

6. Country where you currently live

7. Sector/Profession (choose all that apply)

Check all that apply.

- [ ] Teacher
- [ ] Educational Assistant
- [ ] School Administrator
- [ ] Government
- [ ] Recreation
- [ ] Healthcare
- [ ] Non-Governmental Organization
- [ ] Private Sector
- [ ] Research
- [ ] Parent
- [ ] Student

Other: [ ] ______________________

8. Are you a member of the Sedentary Behaviour Research Network?

Mark only one oval.

- ( ) Yes
- ( ) No

Title, Preamble and Glossary

The Title, Preamble and Glossary are provided below. After reading each section, please indicate your level of agreement with each of the following statements (1 = strongly disagree, 5 = strongly agree).

Title: SBRN School-Related Sedentary Behaviour Recommendations

9. The title is clearly stated.

Mark only one oval.

	1	2	3	4	5	
Strongly Disagree	◯	◯	◯	◯	◯	Strongly Agree

10. I ___________ with how the title is stated.

Mark only one oval.

	1	2	3	4	5	
Strongly Disagree	◯	◯	◯	◯	◯	Strongly Agree

11. Feel free to provide additional comments related to the title.

____

Preamble

Preamble:

These recommendations are applicable for healthy school-aged children and youth attending school irrespective of gender, race, nationality, or socio-economic status. Although they were not the focus of this process, online/distance learners, older and younger students, and those with special needs may also benefit from these recommendations, although they may need to be adapted to meet these students' specific needs.

These recommendations are based on the best available evidence and expert consensus and support a healthy schools approach. They recognize that students benefit from a balance of learning activities that include time spent indoors and outdoors, and a variety of sedentary and movement-based activities each day.

Educators, school administrators, policy makers, parents/guardians, caregivers, physicians, and other healthcare providers should support students to meet these recommendations. The application of these recommendations should be tailored to the personal and developmental needs of individual students.

Further details regarding the background research informing these recommendations, as well as future research directions are available at www.sedentarybehaviour.org.

12. The preamble is clearly stated.

Mark only one oval.

	1	2	3	4	5	
Strongly Disagree	⬭	⬭	⬭	⬭	⬭	Strongly Agree

13. I _________ with how the preamble is stated.

Mark only one oval.

	1	2	3	4	5	
Strongly Disagree	◯	◯	◯	◯	◯	Strongly Agree

14. I would use (e.g. circulate) the preamble.

Mark only one oval.

	1	2	3	4	5	
Strongly Disagree	◯	◯	◯	◯	◯	Strongly Agree

15. Feel free to provide additional comments related to the preamble.

____

____

____

____

____

Glossary

Glossary:

School-related: occurring during school hours (e.g., classroom, recess) or outside of school hours but assigned by the school (e.g., homework).

Sedentary behaviour: any waking behaviour characterized by an energy expenditure ≤1.5 metabolic equivalents (METs), while in a sitting, reclining, or lying posture (Tremblay et al., 2017).

Screen-time: time spent participating in screen-based behaviours, including smartphones, smartboards, tablets, computers, television, and video games. This includes both educational and recreational use of screen-based devices (Tremblay et al., 2017).

Passive screen time: screen-based behaviours that involve little cognitive or physical engagement or participation, such as watching videos while engaging in sedentary behaviour (Hallgren et al., 2020).

Active screen time: screen-based behaviours that require cognitive or physical effort, such as reading, problem-solving or movement (Hallgren et al., 2020).

Homework: an assignment given to students to be completed outside the regular class period (Merriam-Webster, n.d.).

Physical activity: any waking non-sedentary behaviour (e.g. active sitting, standing, walking, playing) (Tremblay et al., 2017).

Movement breaks: a period of any intensity of physical activity (e.g. light, moderate or vigorous) between two periods of sedentary behaviour (Tremblay et al., 2017).

Media-multitasking: using two or more types of screen-based media simultaneously, or using screen-based media while engaging in non-screen-based activities (e.g. texting or using social media while studying or walking) (van der Schuur et al., 2015).

16. The definitions within the glossary are clearly stated.

Mark only one oval.

	1	2	3	4	5	
Strongly Disagree	◯	◯	◯	◯	◯	Strongly Agree

17. I _______ with how the glossary is stated.

Mark only one oval.

	1	2	3	4	5	
Strongly Disagree	◯	◯	◯	◯	◯	Strongly Agree

18. Feel free to provide additional comments related to the glossary.

Recommendations (1)

The draft recommendations are provided below. Please indicate your level of agreement with each of the following statements (1 = strongly disagree, 5 = strongly agree).

Draft Recommendations

A healthy school-day includes:

- Breaking up periods of extended sedentary behaviour with regularly scheduled movement breaks:
  - At least once every 30 minutes for ages 5-11 years.
  - At least once every hour for ages 12-18 years.

- Limiting sedentary homework to no more than 10 minutes per night, per grade level (e.g., no more than 10 minutes per night for grade 1, no more than 60 minutes per night for grade 6, and no more than 120 minutes per night for grade 12).

- Regardless of the location, school-related screen use should be meaningful, mentally or physically active, and serve a specific pedagogical purpose that enhances learning. Passive screen time should not be a routine part of learning.

- When screen-based learning is warranted:
  - Limit time on devices, especially for students 5-11 years of age.
  - Take a device break at least once every 20 minutes.
  - Discourage media-multitasking in the classroom and while doing homework.

Consider replacing sedentary learning activities with movement-based learning activities and replacing screen-based learning activities with non-screen-based learning activities.

19. The recommendations are clearly stated.

Mark only one oval.

	1	2	3	4	5	
Strongly Disagree	◯	◯	◯	◯	◯	Strongly Agree

20. I _______ with how the recommendations are stated.

Mark only one oval.

	1	2	3	4	5	
Strongly Disagree	◯	◯	◯	◯	◯	Strongly Agree

21. The recommendations are realistic and achievable for educators.

Mark only one oval.

	1	2	3	4	5	
Strongly Disagree	◯	◯	◯	◯	◯	Strongly Agree

22. The recommendations are realistic and achievable for parents.

Mark only one oval.

	1	2	3	4	5	
Strongly Disagree	◯	◯	◯	◯	◯	Strongly Agree

23. The recommendations are realistic and achievable for students.

Mark only one oval.

	1	2	3	4	5	
Strongly Disagree	◯	◯	◯	◯	◯	Strongly Agree

24. The recommendations are realistic and achievable for school administrators.

Mark only one oval.

	1	2	3	4	5	
Strongly Disagree	◯	◯	◯	◯	◯	Strongly Agree

25. The recommendations are appropriately specific.

Mark only one oval.

	1	2	3	4	5	
Strongly Disagree	◯	◯	◯	◯	◯	Strongly Agree

26. The recommendations will be useful in my professional life.

Mark only one oval.

	1	2	3	4	5	
Strongly Disagree	◯	◯	◯	◯	◯	Strongly Agree

27. The recommendations will be useful in my personal life.

Mark only one oval.

	1	2	3	4	5	
Strongly Disagree	◯	◯	◯	◯	◯	Strongly Agree

28. Feel free to provide any additional comments related to the recommendations.

Recommendations (2)

Please respond to the following questions related to the importance, use, and costs/benefits of the recommendations.

Draft Recommendations

A healthy school-day includes:

- Breaking up periods of extended sedentary behaviour with regularly scheduled movement breaks:
  - At least once every 30 minutes for ages 5-11 years.
  - At least once every hour for ages 12-18 years.

- Limiting sedentary homework to no more than 10 minutes per night, per grade level (e.g., no more than 10 minutes per night for grade 1, no more than 60 minutes per night for grade 6, and no more than 120 minutes per night for grade 12).

- Regardless of the location, school-related screen use should be meaningful, mentally or physically active, and serve a specific pedagogical purpose that enhances learning. Passive screen time should not be a routine part of learning.

- When screen-based learning is warranted:
  - Limit time on devices, especially for students 5-11 years of age.
  - Take a device break at least once every 20 minutes.
  - Discourage media-multitasking in the classroom and while doing homework.

Consider replacing sedentary learning activities with movement-based learning activities and replacing screen-based learning activities with non-screen-based learning activities.

29. How important are these recommendations to your professional work?

Mark only one oval.

	1	2	3	4	5	
Not at all						Very

30. Feel free to provide additional comments related to the importance of these recommendations.

31. How relevant are these recommendations to your professional work?

Mark only one oval.

	1	2	3	4	5	
Not at all	◯	◯	◯	◯	◯	Very

32. Feel free to provide additional comments related to the relevance of these recommendations.

33. How often would you use these recommendations in your professional work?

Mark only one oval.

	1	2	3	4	5	
Never	◯	◯	◯	◯	◯	Always

34. How easy or difficult would you find using these recommendations?

Mark only one oval.

	1	2	3	4	5	
Very Difficult	◯	◯	◯	◯	◯	Very Easy

35. Feel free to provide additional comments related to the usage of these recommendations.

____

____

____

____

____

36. The costs (e.g. time, financial, opportunity) for you or your organization to implement these recommendations are likely to be small or negligible compared to not implementing the recommendations.

Mark only one oval.

- ◯ I don't know
- ◯ Strongly Disagree
- ◯ Somewhat Disagree
- ◯ Neither Agree Nor Disagree
- ◯ Somewhat Agree
- ◯ Strongly Agree
- ◯ Not Applicable

37. The benefits of using these recommendations are likely to outweigh the costs (e.g. time, financial, opportunity) in your professional work.

Mark only one oval.

- I don't know
- Strongly Disagree
- Somewhat Disagree
- Neither Agree Nor Disagree
- Somewhat Agree
- Strongly Agree
- Not Applicable

38. Feel free to provide additional comments related to the costs/benefits of implementing these recommendations.

39. Following these recommendations are likely to benefit students regardless of gender, race, ethnicity, nationality or socioeconomic status.

Mark only one oval.

	1	2	3	4	5	
Strongly Disagree						Strongly Agree

40. Feel free to provide additional comments related to the benefits of these recommendations for different demographic groups.

41. Please provide at least one example of how you would implement (or apply) these recommendations in your current profession.

Implementation Strategies

Suggested implementation strategies are provided below. After reading each section, please indicate your level of agreement with each of the following statements (1 = strongly disagree, 5 = strongly agree).

Implementation Strategies

How to Implement These Recommendations[1]:

Educators, school administrators, policy makers, parents/guardians, caregivers, physicians, and other healthcare providers can implement these recommendations using the four "Ms" approach:

- **Manage** sedentary behaviour and screen use.
  - See recommendations above.

- Encourage **meaningful** screen use.
  - Prioritize face-to-face interactions over screen use.
  - Use screens for specific purposes, when they are the best pedagogical tool for the job and are likely to enhance learning.
  - Use screens for mental and physical engagement, rather than passive viewing.
  - Turn screens off when not in use, including background TV or videos.
  - Avoid using screens as the default method for content delivery or classroom management.
  - Encourage students to review and self-regulate their screen use, and plan time for outdoor play and physical activity.

- **Model** healthy screen use
  - Avoid screen-use during meal and snack times.
  - Minimize screen-based multitasking, and do not text while driving.
  - Discourage screen-based homework within an hour of bedtime

- **Monitor** for signs of problematic screen use.
  - Complaints about being bored or unhappy without access to technology.
  - Oppositional behaviour in response to screen use limits
  - Screen use that interferes with family activities, sleep, physical activity, offline play, school or face-to-face interactions
  - Negative emotions following time spent playing video games, texting, or using social media.
  - Follow-up with your health care provider if concerned about screen-based behaviours.

[1]Adapted with permission from the Canadian Pediatric Society (Canadian Paediatric Society, 2017).

42. The implementation strategies are clearly stated.

Mark only one oval.

	1	2	3	4	5	
Strongly Disagree	◯	◯	◯	◯	◯	Strongly Agree

43. I _______ with how the implementation strategies are stated.

Mark only one oval.

	1	2	3	4	5	
Strongly Disagree						Strongly Agree

44. The implementation strategies are realistic and achievable for educators.

Mark only one oval.

	1	2	3	4	5	
Strongly Disagree						Strongly Agree

45. The implementation strategies are realistic and achievable for parents.

Mark only one oval.

	1	2	3	4	5	
Strongly Disagree						Strongly Agree

46. The implementation strategies are realistic and achievable for students.

Mark only one oval.

	1	2	3	4	5	
Strongly Disagree						Strongly Agree

47. The implementation strategies are realistic and achievable for school administrators.

Mark only one oval.

	1	2	3	4	5	
Strongly Disagree	◯	◯	◯	◯	◯	Strongly Agree

48. The implementation strategies are realistic and achievable for physicians and other healthcare providers.

Mark only one oval.

	1	2	3	4	5	
Strongly Disagree	◯	◯	◯	◯	◯	Strongly Agree

49. The implementation strategies will be useful in my professional life.

Mark only one oval.

	1	2	3	4	5	
Strongly Disagree	◯	◯	◯	◯	◯	Strongly Agree

50. The implementation strategies will be useful in my personal life.

Mark only one oval.

	1	2	3	4	5	
Strongly Disagree	◯	◯	◯	◯	◯	Strongly Agree

51. Feel free to provide additional comments related to the implementation strategies.

Thank you.

Thank you for your feedback on these draft recommendations. Feel free to share any additional comments in the box below.

52. Additional comments.

This content is neither created nor endorsed by Google.

Google Forms
