# Supplementary material for: International school-related sedentary behaviour recommendations for children and youth
Source: Int J Behav Nutr Phys Act. 2022 Apr 5;19:39. doi: 10.1186/s12966-022-01259-3 (PMC8979784; doi:10.1186/s12966-022-01259-3)
Supplement: Supplementary file 6 — Additional file 6: S6. Stakeholder Feedback Responses. [file 12966_2022_1259_MOESM6_ESM.docx]

**SBRN School-Related Sedentary Behaviour Recommendations Public Consultation**

**Summary of Survey Responses**

Working with a team of international experts, the Sedentary Behaviour Research Network (SBRN) has developed evidence-based school-related sedentary behaviour recommendations and disseminated a **survey** to gather feedback from stakeholders on the draft version of these recommendations. The survey was live for 2 and a half weeks, from July 9th to 26th, 2021, and was completed by 148 respondents from 23 countries. Of these, 59 (39.9%) identified as SBRN members. The majority of survey participants were females (64%), aged 25 to 64 years (95.9%), and currently living in Canada (54.7%). The most represented occupational groups among respondents were researchers (45.3%), teachers (30.4%), and health care professionals (15.5%) (respondents could select more than 1).

When asked about their level of **agreement with the title, preamble, and glossary**, the majority of participants responded “strongly agree” or “somewhat agree” that the title is clearly stated (86.3%) and with how it is stated (80%), that the preamble is clearly stated (89.2%) and with how it is stated (83%), and that the glossary is clearly stated (88.5%) and with how it is stated (88.4%).

Regarding the **agreement with draft recommendations/statements**, the majority of respondents “strongly agree” or “somewhat agree” that the recommendations are clearly stated (90.5%) and with how they are stated (84.4%). Contrarily, a relatively small percent of respondents reported that the draft recommendations are not clearly stated (2.7%) and “somewhat disagree” with how they are stated (4.1%). The majority of survey participants felt that the recommendations are appropriately specific (77.2%) and realistic and achievable for educators (65.3%), students (63.5%), school administrators (63.4%), and parents (62.2%). Of the respondents, 65.8% and 66% indicated that the recommendations will be useful in their professional and personal life, respectively.

Regarding the **importance, use, and costs/benefits of the recommendations**, the majority of survey participants consider them “very important” or “somewhat important” (78.2%) and “very relevant” or “somewhat relevant” (78.2%) to their professional work. Among the survey participants, the majority reported that they would use these recommendations in their professional work “always” or “very often” (60%) and find these recommendations “very easy” or “easy” (57.6%) to use. Of the participants, 43.2% reported they “strongly agree” or “somewhat agree” that the costs (e.g. time, financial, opportunity) for them or their organization to implement these recommendations would likely be small or negligible compared to not implementing them, while 12.3% disagreed with that. The majority of respondents (67.8%) felt that the benefits of using these recommendations are likely to outweigh the costs in their professional work. Following these recommendations are likely to benefit students regardless of gender, race, ethnicity, nationality, or socioeconomic status, as indicated by 87% of respondents.

In terms of the level of **agreement with proposed implementation strategies,** the majority of participants responded “strongly agree” or “somewhat agree” that the implementation strategies are clearly stated (93.1%) and with how they are stated (89%). The respondents consider the proposed implementation strategies to be realistic and achievable for educators (75.2%), physicians and other healthcare providers (75%), school administrators (73.8%), students (72.8%), and parents (70%). Of the survey participants, 76.2% and 75.3% indicated that the proposed implementation strategies will be useful in their professional and personal life, respectively.

Overall, the survey demonstrated a high level of agreement, though not unanimous, with the draft school-related sedentary behaviour recommendations developed by SBRN.

*****

A more detailed summary of the survey findings is below.

Total number of respondents: 148

Completion period: from 09/07/2021 to 26/07/2021

| **General information about respondents,** number of responses (%) | | |
| --- | --- | --- |
| 1 | Age of respondents | Total responses N=146   - 25-64 years – 140 (95.9%) - 65 years and over – 4 (2.7%) - 16-24 years – 1 (0.7%) - “?” – 1 (0.7%) |
| 2 | Gender of respondents | Total responses N=147   - Female – 94 (64%) - Male – 50 (34%) - Prefer not to say – 3 (2%) - Non-binary – 0 |
| 3 | Language spoken most frequently at home | Total responses N=145  Total languages=22   - English – 102 (70.4%) - French – 9 (6.2%) - Chinese – 7 (4.8%) - Other – 27 (18.6%) |
| 4 | Nationality | Total responses N=145  Total nationalities represented=28   - Canadian – 77 (53.1%) - Australian – 10 (6.9%) - Kenyan – 8 (5.5%) - Other – 50 (34.5%) |
| 5 | Country where respondents currently live | Total responses N=148  Total countries represented=23   - Canada – 81 (54.7%) - Australia – 15 (10.1%) - Kenya – 8 (5.4%) - Other – 44 (29.7%) |
| 6 | Sector/profession | Total responses N=148  (respondents could select more than 1 answer)   - Teacher – 45 (30.4%) - Educational Assistant – 4 (2.7%) - School Administrator – 4 (2.7%) - Government – 20 (13.5%) - Recreation – 10 (6.8%) - Healthcare – 23 (15.5%) - Non-Governmental organization – 6 (4.1%) - Private Sector – 4(2.7%) - Research – 67 (45.3%) - Parent – 15 (10.1%) - Student – 11 (7.4%) - Other – 12 (8.1%) |
| 7 | SBRN member | Total responses N=148   - Yes – 59 (39.9%) - No – 89 (60.1%) |
| **Agreement with the Title, Preamble, and Glossary,** number of responses (%) | | |
| 8 | The **title** is clearly stated. | Total responses N=146   - 1-Strongly disagree – 2 (1.4%) - 2-Somewhat disagree – 7 (4.8%) - 3-Neither agree nor disagree – 11 (7.5%) - 4-Somewhat agree – 47 (32.2%) - 5-Strongly agree – 79 (54.1%) |
| 9 | I _____________ with how the title is stated. | Total responses N=145   - 1-Strongly disagree – 3 (2.1%) - 2-Somewhat disagree – 2 (1.4%) - 3-Neither agree nor disagree - 24 (16.6%) - 4-Somewhat agree – 55 (37.9%) - 5-Strongly agree – 61 (42.1%) |
| 10 | Additional comments related to the title | Total responses N=57  Most common comments (N=19) - related to the acronym “SBRN” (e.g., remove, spell it out, move to the end of the title). |
| 11 | The **preamble** is clearly stated. | Total responses N=148   - 1-Strongly disagree – 3 (2%) - 2-Somewhat disagree – 4 (2.7%) - 3-Neither agree nor disagree – 9 (6.1%) - 4-Somewhat agree – 52 (35.1%) - 5-Strongly agree – 80 (54.1%) |
| 12 | I _____________ with how the preamble is stated. | Total responses N=147   - 1-Strongly disagree – 3 (2%) - 2-Somewhat disagree – 2 (1.4%) - 3-Neither agree nor disagree – 20 (13.6%) - 4-Somewhat agree – 53 (36.1%) - 5-Strongly agree – 69 (46.9%) |
| 13 | I would use (e.g. circulate) the preamble. | Total responses N=147   - 1-Strongly disagree – 6 (4.2%) - 2-Somewhat disagree – 5 (3.5%) - 3-Neither agree nor disagree – 23 (16.1%) - 4-Somewhat agree – 50 (35%) - 5-Strongly agree – 59 (41.3%) |
| 14 | Additional comments related to the preamble | Total responses N=21  Most common comments (N=6) - related to using the word “healthy” (e.g., not clear what it means, not inclusive, remove it) |
| 15 | The definitions within the **glossary** are clearly stated. | Total responses N=148   - 1-Strongly disagree – 1 (0.7%) - 2-Somewhat disagree – 6 (4.1%) - 3-Neither agree nor disagree – 10 (6.8%) - 4-Somewhat agree – 41 (27.7%) - 5-Strongly agree – 90 (60.8%) |
| 16 | I ________ with how the glossary is stated. | Total responses N=147   - 1-Strongly disagree – 2 (1.4%) - 2-Somewhat disagree – 4 (2.7%) - 3-Neither agree nor disagree – 11 (7.5%) - 4-Somewhat agree – 47 (32%) - 5-Strongly agree – 83 (56.5%) |
| 17 | Additional comments related to the glossary | Total responses N=30   - Most common comments (N=8) - suggestions to use simpler language (e.g., avoid/better describe terms like MET) |
| **Agreement with the Draft Recommendations,** number of responses (%) | | |
| 18 | The **recommendations** are clearly stated. | Total responses N=148   - 1-Strongly disagree – 0 (0%) - 2-Somewhat disagree – 4 (2.7%) - 3-Neither agree nor disagree – 10 (6.8%) - 4-Somewhat agree – 37 (25%) - 5-Strongly agree – 97 (65.5%) |
| 19 | I ________ with how the recommendations are stated. | Total responses N=147   - 1-Strongly disagree – 0 (0%) - 2-Somewhat disagree – 6 (4.1%) - 3-Neither agree nor disagree – 17 (11.6%) - 4-Somewhat agree – 48 (32.7%) - 5-Strongly agree – 76 (51.7%) |
| 20 | The recommendations are realistic and achievable for **educators**. | Total responses N=144   - 1-Strongly disagree – 5 (3.5%) - 2-Somewhat disagree – 14 (9.7%) - 3-Neither agree nor disagree – 31 (21.5%) - 4-Somewhat agree – 55 (38.2%) - 5-Strongly agree – 39 (27.1%) |
| 21 | The recommendations are realistic and achievable for **parents**. | Total responses N=148   - 1-Strongly disagree – 4 (2.7%) - 2-Somewhat disagree – 14 (9.5%) - 3-Neither agree nor disagree – 38 (25.7%) - 4-Somewhat agree – 51 (34.5%) - 5-Strongly agree – 41 (27.7%) |
| 22 | The recommendations are realistic and achievable for **students**. | Total responses N=145   - 1-Strongly disagree – 3 (2.1%) - 2-Somewhat disagree – 15 (10.3%) - 3-Neither agree nor disagree – 35 (24.1%) - 4-Somewhat agree – 51 (35.2%) - 5-Strongly agree – 41 (28.3%) |
| 23 | The recommendations are realistic and achievable for **school administrators**. | Total responses N=142   - 1-Strongly disagree – 2 (1.4%) - 2-Somewhat disagree – 11 (7.7.%) - 3-Neither agree nor disagree – 39 (27.5%) - 4-Somewhat agree – 50 (35.2%) - 5-Strongly agree – 40 (28.2%) |
| 24 | The recommendations are appropriately **specific**. | Total responses N=145   - 1-Strongly disagree – 2 (1.4%) - 2-Somewhat disagree – 10 (6.9%) - 3-Neither agree nor disagree – 21 (14.5%) - 4-Somewhat agree – 54 (37.2%) - 5-Strongly agree – 58 (40%) |
| 25 | The recommendations will be **useful** **in my professional life**. | Total responses N=146   - 1-Strongly disagree – 4 (2.7%) - 2-Somewhat disagree – 13 (8.9%) - 3-Neither agree nor disagree – 33 (22.6%) - 4-Somewhat agree – 41 (28.1%) - 5-Strongly agree – 55 (37.7%) |
| 26 | The recommendations will be **useful in my personal life.** | Total responses N=147   - 1-Strongly disagree – 7(4.8%) - 2-Somewhat disagree – 12 (8.2%) - 3-Neither agree nor disagree – 31 (21.1%) - 4-Somewhat agree – 40 (27.2%) - 5-Strongly agree – 57 (38.8%) |
| 27 | Additional comments related to the recommendations | Total comments N=70   - Most common (N=11) – suggestions to better describe movement breaks (e.g., specify recommended duration, give examples) |
| **Importance, use, and costs/benefits of the recommendations**, number of responses (%) | | |
| 28 | How **important** are these recommendations to your professional work? | Total responses N=147   - 1-Not at all important – 1 (0.7%) - 2-Not very important – 8 (5.4%) - 3-Neutral – 23 (15.6%) - 4-Important – 60 (40.8%) - 5-Very important – 55 (37.4%) |
| 29 | Additional comments related to the importance of these recommendations. | Total comments N=38   - Most common (N=8) – stating that the recommendations are very helpful/important |
| 30 | How **relevant** are these recommendations to your professional work? | Total responses N=147   - 1-Not at all relevant – 0 (0%) - 2-Not very relevant – 9 (6.1%) - 3-Neutral – 23 (15.6%) - 4-Relevant – 59 (40.1%) - 5-Very relevant – 56 (38.1%) |
| 31 | Additional comments related to the relevance of these recommendations | Total comments N=21   - A mix of general comments on the relevance to respondents’ professional life (e.g., of great relevance, not very relevant) |
| 32 | **How often** would you use these recommendations in your professional work? | Total responses N=145   - 1-Never – 2 (1.4%) - 2-Rarely – 14 (9.7%) - 3-Sometimes – 42 (29%) - 4-Very often – 58 (40%) - 5-Always – 29 (20%) |
| 33 | **How easy or difficult** would you find using these recommendations? | Total responses N=144   - Very difficult – 4 (2.8%) - Difficult – 16 (11.1%) - Neither difficult nor easy – 41 (28.5%) - Easy – 53 (36.8%) - Very easy – 30 (20.8%) |
| 34 | Additional comments related to the usage of these recommendations. | Total comments N=33   - Most common (N=4) – suggestions to simplify/improve clarity to help with usage |
| 35 | The **costs** (e.g. time, financial, opportunity) for you or your organization to implement these recommendations are likely to be small or negligible compared to not implementing the recommendations. | Total responses N=146   - I don’t know – 19 (13%) - Strongly disagree – 5 (3.4%) - Somewhat disagree – 13 (8.9%) - Neither agree nor disagree – 17 (11.6%) - Somewhat agree – 30 (20.5%) - Strongly agree – 33 (22.6%) - Not applicable – 29 (19.9%) |
| 36 | The **benefits** of using these recommendations are likely to outweigh the costs (e.g. time, financial, opportunity) in your professional work. | Total responses N=146   - I don’t know – 16 (11%) - Strongly disagree – 0 (0%) - Somewhat disagree – 4 (2.7%) - Neither agree nor disagree – 10 (6.8%) - Somewhat agree – 34 (23.3%) - Strongly agree – 65 (44.5%) - Not applicable – 17 (11.6%) |
| 37 | Additional comments related to the costs/benefits of implementing these recommendations | Total comments N=14   - Most common (N=3) - “it depends”/”difficult to answer” |
| 38 | Following these recommendations are likely to benefit students regardless of gender, race, ethnicity, nationality, or socioeconomic status. | Total responses N=146   - Strongly disagree – 3 (2.1%) - Somewhat disagree – 1 (0.7%) - Neither agree nor disagree – 15 (10.3%) - Somewhat agree – 47 (32.2%) - Strongly agree – 80 (54.8%) |
| 39 | Additional comments related to the benefits of these recommendations for different demographic groups. | Total comments N=28   - Most common (N=4) – different access to resources may have an impact (e.g., safe play area, screens/internet) |
| 40 | Please provide at least one example of how you would implement (or apply) these recommendations in your current profession. | Total responses N=82   - Research (e.g., questionnaire development, use as benchmarks, reference in academic papers, implementation research) - Teaching (e.g., use for lessons planning, inform students) - Healthcare (e.g., patients and parents counseling) - Policy (e.g., as an advocacy tool for policy changes, incorporate into policy) - Raising awareness (e.g., dissemination via social media, training for teachers) |
| **Agreement with Implementation Strategies,** number of responses (%) | | |
| 41 | The implementation strategies are clearly stated. | Total responses N=145   - Strongly disagree – 2 (1.4%) - Somewhat disagree – 2 (1.4%) - Neither agree nor disagree – 6 (4.1%) - Somewhat agree – 51 (35.2%) - Strongly agree – 84 (57.9%) |
| 42 | I ________ with how the implementation strategies are stated. | Total responses N=145   - Strongly disagree – 1 (0.7%) - Somewhat disagree – 6 (4.1%) - Neither agree nor disagree – 9 (6.2%) - Somewhat agree – 53 (36.6%) - Strongly agree – 76 (52.4%) |
| 43 | The implementation strategies are realistic and achievable for **educators**. | Total responses N=145   - Strongly disagree – 4 (2.8%) - Somewhat disagree – 6 (4.1%) - Neither agree nor disagree – 26 (17.9%) - Somewhat agree – 65 (44.8%) - Strongly agree – 44 (30.3%) |
| 44 | The implementation strategies are realistic and achievable for **parents**. | Total responses N=147   - Strongly disagree – 3 (2%) - Somewhat disagree – 6 (4.1%) - Neither agree nor disagree – 35 (23.8%) - Somewhat agree – 69 (46.9%) - Strongly agree – 34 (23.1%) |
| 45 | The implementation strategies are realistic and achievable for **students**. | Total responses N=147   - Strongly disagree – 3 (2%) - Somewhat disagree – 10 (6.8%) - Neither agree nor disagree – 27 (18.4%) - Somewhat agree – 74 (50.3%) - Strongly agree – 33(22.4%) |
| 46 | The implementation strategies are realistic and achievable **for school administrators**. | Total responses N=145   - Strongly disagree – 3 (2.1%) - Somewhat disagree – 6 (4.1%) - Neither agree nor disagree – 29 (20%) - Somewhat agree – 63 (43.4%) - Strongly agree – 44 (30.3%) |
| 47 | The implementation strategies are realistic and achievable for **physicians and other healthcare providers**. | Total responses N=144   - Strongly disagree – 4 (2.8%) - Somewhat disagree – 4 (2.8%) - Neither agree nor disagree – 28 (19.4%) - Somewhat agree – 67 (46.5%) - Strongly agree – 41 (28.5%) |
| 48 | The implementation strategies will be **useful in my professional life**. | Total responses N=143   - Strongly disagree – 3 (2.1%) - Somewhat disagree – 8 (5.6%) - Neither agree nor disagree – 23 (16.1%) - Somewhat agree – 54 (37.8%) - Strongly agree – 55 (38.5%) |
| 49 | The implementation strategies will be **useful in my personal life**. | Total responses N=146   - Strongly disagree – 4 (2.7%) - Somewhat disagree – 7 (4.8%) - Neither agree nor disagree – 25 (17.1%) - Somewhat agree – 50 (34.2%) - Strongly agree – 60 (41.1%) |
| 50 | Additional comments related to the implementation strategies. | Total comments N=35   - Common comments (N=5) - related to the inclusion of “do not text while driving” (e.g., not appropriate/ not relevant here) |
| **Additional Comments** | | |
| 51 | Any other comments | Total comments N=27   - Common comments (N=17) – positive feedback (e.g., thank you, great initiative, looking forward to releasing the recommendations) |
