# Supplementary figures and images for: International school-related sedentary behaviour recommendations for children and youth
Source: Int J Behav Nutr Phys Act. 2022 Apr 5;19:39. doi: 10.1186/s12966-022-01259-3 (PMC8979784; doi:10.1186/s12966-022-01259-3)

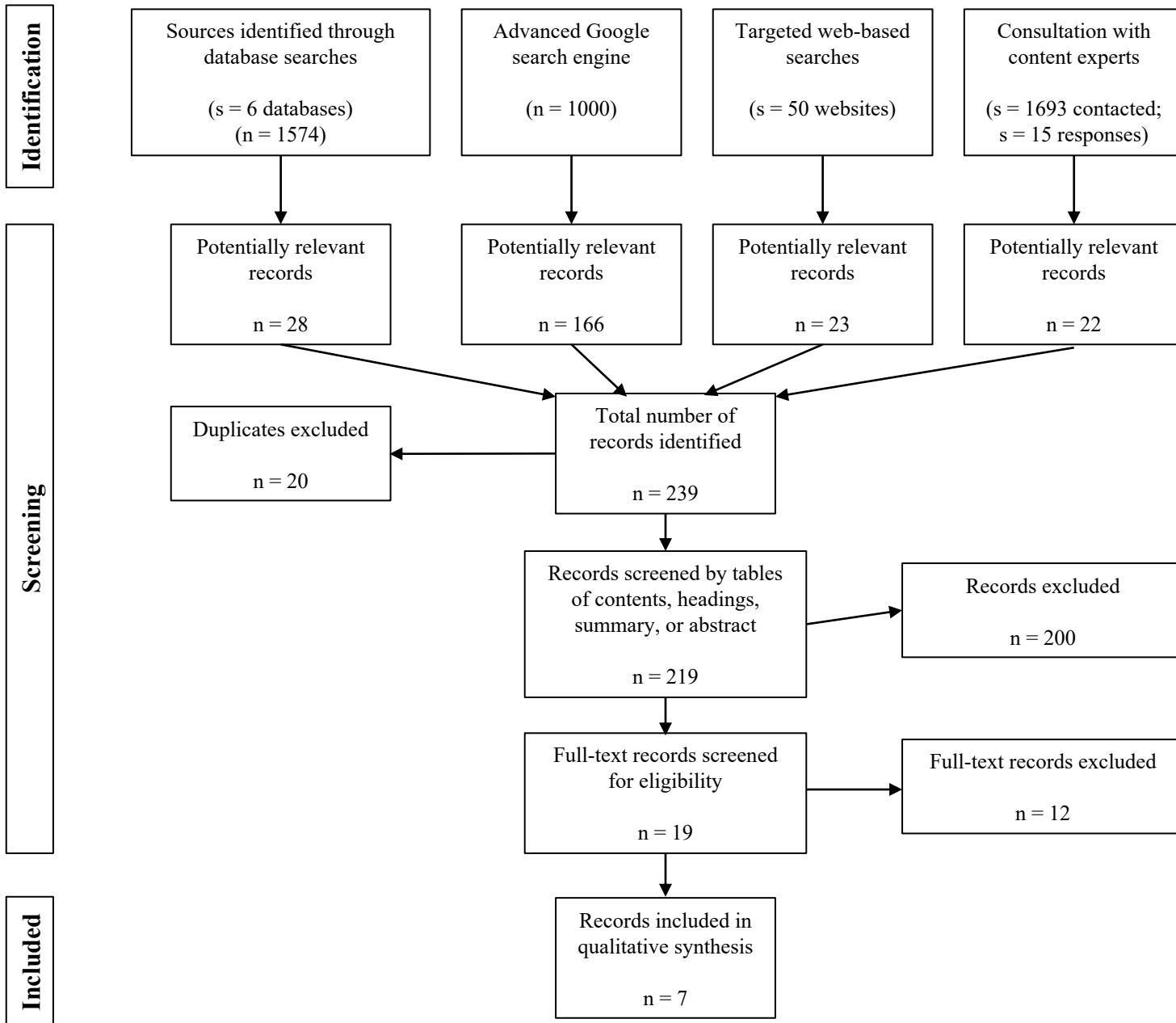

Supplement: Supplementary file 7 — Additional file 7: S7. Environmental Scan Flow Diagram. [file 12966_2022_1259_MOESM7_ESM.pdf]
