# Supplementary material for: International school-related sedentary behaviour recommendations for children and youth
Source: Int J Behav Nutr Phys Act. 2022 Apr 5;19:39. doi: 10.1186/s12966-022-01259-3 (PMC8979784; doi:10.1186/s12966-022-01259-3)
Supplement: Supplementary file 8 — Additional file 8: S8. Characteristics of Included Guidelines. [file 12966_2022_1259_MOESM8_ESM.docx]

| Document | Issuing authority/ organization; type | Location | Year | Recommendations | Guideline Quality | URL |
| --- | --- | --- | --- | --- | --- | --- |
| Health & Safety Best Practice Guidelines: Digital Devices | Maryland Dept. of Health/State Dept. of Education; P/S government organization | Maryland, USA | 2019 | General recommendations for digital device use | High | http://marylandpublicschools.org/programs/Documents/ITSLM/Health_and_Safety_Best_Practice_Guidelines_Digital_Devices.pdf |
| Guidelines for Digital Education | Dept. of School Education & Literacy, Ministry of Human Res. Development; national government | India | 2020 | Screen time limits for online classes/digital education | High | https://www.education.gov.in/en/pragyata-guidelines-digital-education |
| Suggested Strategies in Implementing Distance Learning Delivery Modalities for School Year 2020-2021 | Dept. of Education; national government | Philippines | 2020 | Screen time limits for online classes/digital education | High | https://depeddasma.edu.ph/um-suggested-strategies-in-implementing-distance-learning-delivery-modalities-dldm-for-school-year-2020-2021/ |
| Daily Physical Activity in Elementary Schools, Grades 1-8 | Ministry of Education; P/S government organization | Ontario, Canada | 2017 | Limiting/reducing sedentary behaviour and breaking up long periods of sedentary time | High | http://www.edu.gov.on.ca/eng/healthyschools/dpa.html |
| Guidelines for PA in State Schools | Queensland Gov., Dept. of Education; P/S government organization | Queensland, Australia | 2019 | Limiting/reducing sedentary behaviour and breaking up long periods of sedentary time | High | https://education.qld.gov.au/students/student-health-safety-wellbeing/student-health/physical-activity-ss |
| WHO HBSC Report on SB | WHO and HBSC’s PA Focus Group; United Nations specialized agency | International | Not stated | Limiting/reducing sedentary behaviour and breaking up long periods of sedentary time | High | http://www.hbsc.org/news/index.aspx?ni=2516 |
| Digital Devices in the Classroom - Health & Safety Guidelines | Virginia Dept. of Education/Dept. of Health; P/S government organization | Virginia, USA | 2021-2022 school year | Digital device use in the classroom/at home learning, screen time/break frequency, and movement/activity | High | https://www.doe.virginia.gov/instruction/virtual_learning/digital-devices-infographic.pdf |

Table S4. Characteristics of included school-related sedentary behaviour and screen time guidelines.
